# Mask Detection and Social Distance Identification Using Internet of Things and Faster R-CNN Algorithm

**DOI:** 10.1155/2022/2103975

**Published:** 2022-02-01

**Authors:** S. Meivel, Nidhi Sindhwani, Rohit Anand, Digvijay Pandey, Abeer Ali Alnuaim, Alaa S. Altheneyan, Mohamed Yaseen Jabarulla, Mesfin Esayas Lelisho

**Affiliations:** ^1^M. Kumarasamy College of Engineering, Karur, Tamil Nadu, India; ^2^AIIT, Amity University, Noida, India; ^3^DSEU, G. B. Pant Okhla-1 Campus, New Delhi, India; ^4^Department of Technical Education, IET Lucknow, Dr. A. P. J Abdul Kalam Technical University Lucknow, Lucknow, India; ^5^Department of Computer Science and Engineering, College of Applied Studies and Community Services, King Saud University, P.O. Box 22459, Riyadh 11495, Saudi Arabia; ^6^School of Electrical Engineering and Computer Science, Gwangju Institute of Science and Technology, Republic of Korea; ^7^Department of Statistics, College of Natural and Computational Science, Mizan-Tepi University, Tepi, Ethiopia

## Abstract

The drones can be used to detect a group of people who are unmasked and do not maintain social distance. In this paper, a deep learning-enabled drone is designed for mask detection and social distance monitoring. A drone is one of the unmanned systems that can be automated. This system mainly focuses on Industrial Internet of Things (IIoT) monitoring using Raspberry Pi 4. This drone automation system sends alerts to the people via speaker for maintaining the social distance. This system captures images and detects unmasked persons using faster regions with convolutional neural network (faster R-CNN) model. When the system detects unmasked persons, it sends their details to respective authorities and the nearest police station. The built model covers the majority of face detection using different benchmark datasets. OpenCV camera utilizes 24/7 service reports on a daily basis using Raspberry Pi 4 and a faster R-CNN algorithm.

## 1. Introduction

The coronavirus disease 2019 (COVID-19) epidemic has arisen as a major menace all around the world. As the number of cases is gradually increasing day by day, the government has several difficulties in controlling the pandemic situation. The communication of this disease can only be lessened with the proper collaboration of people. Physical distancing, repeated hand sanitizing, and face masking have proven to be quite efficient to control the spreading of the virus, but everyone is not obeying the guidelines. Various technologies like machine learning (ML) algorithms [1], artificial intelligence (AI) approaches [1], Internet of things (IoT) [2–5], and unmanned aerial vehicles (UAV) [6] give a real-time scenario at any given point about (i) the number of people following physical distancing and (ii) whether people are wearing masks or not.

In today's scenario, COVID-19 [3, 5] has come to be one of the most important topics that ought to be confronted properly. Therefore, there is a requirement to develop an approach that can tackle the issues like unmasked persons and nonmaintenance of physical distancing. With the major developments in the area of industrial IoT (IIoT), remote monitoring can be done with much more ease. The key concept at the back of IoT is to interconnect the different automated components throughout a network. In order to allow the data communication between them, each object is assigned with a different identity and the approach is to design a facial mask detection system based on IIoT.

As of January 20, 2021, the deadly COVID-19 illness, which has affected more than 200 countries and territories, including two international means of transport, has so far caused 96.1 million infections and 2.06 million fatalities globally. Even while there was a shortage of active pharmaceutical experts, there was also a lack of public opposition to COVID-19, making the populace even more vulnerable. The World Health Organization has labeled it a pandemic [7]. Because this is an epidemic with no cure, the only strategy left is to wear a mask. The fact that a face mask may prevent the spread of COVID-19 has led to an increase in its use among the general population. In order to stop the virus from spreading further, the global society must consider quarantine, as well as increasing the social barrier between infected and uninfected individuals. This face mask test is used to make sure that the individual is protected against infection with the airborne virus. Whenever someone coughs, talks, or sneezes, viruses will fly into the air, increasing the risk of spreading disease to their surroundings. To reduce the transmission of disease, infection control specialists use a number of measures, including surgical masks, to ward against contamination.

The main ways in which this paper makes its mark are as follows. A face mask recognition method is used based on quicker R-CNN and YOLO models [8]. To get a full understanding of the main issues involved in face mask detection, something may help in the future when it comes to developing the new face mask detectors. This transfer learning model includes the new information for developing algorithms. It is a machine learning strategy in which the computer learns the skills from a single task and then may apply these skills to other situations. The use of pretrained models as the base point in AI activities, especially those with computational complexity and time concerns, is an emerging trend. The authors used two algorithms, YOLOv3 and accelerated R-CNN, for forecasting, and their results have been compared. Since the R-CNN [8] uses two networks, the first of which is the region proposal network (RPN) [8] which makes recommendations to identify objects, it is more efficient. Each area box is assigned a value by RPN. YOLO is a sophisticated, real-time CNN that identifies objects. The procedure slices the image into regions and guesses where to train these models but it is difficult because of the camera angles and mask types in pictures. Another difficulty that the authors found was the absence of a large dataset with both masked and unmasked categories. The writers had to generate a fresh dataset and use transfer learning to finish the research because of this issue. The social distancing and mask detection are performed on drone footage using artificial intelligence and the faster R-CNN algorithm. The captured images are analyzed using datasets and trained models.

The proposed system identifies only the stored faces in the database. Upon detection of an unmasked face, it sends an e-mail containing the image of the person. In order to design the facemask recognition system, a setup containing Raspberry Pi 4 and an OpenCV camera is used. Raspberry Pi uses Raspbian Stretch as an operating system. In order to train the database of images, a huge number of images at different angles and lighting environments would be collected. A faster R-CNN algorithm is used here to detect facial mask recognition. The current research aims to provide the information to the user using open source technology that comprises faster R-CNN, IIoT, Raspberry Pi 4, OpenCV camera, and UAV. Most of the existing models are not tested in real time. However, the proposed model is verified in real time by embedding the built model on a drone. The faster R-CNN model is used for detecting faces and people's activities. Live social distancing is measured using YOLOv3.

The paper is organized as follows: Section 2 discusses the survey of the existing work related to the proposed work.  Section 3 discusses the proposed system model and the proposed methodology.  Section 4 discusses the results and analysis section in terms of various parameters. At last, the conclusion is drawn.

## 2. Related Work

People are finding many new methods to secure themselves from the COVID-19 pandemic. The researchers working in the fields such as Internet of Things, machine learning, computer vision, and Blockchain are working on the various techniques that can secure and treat the people against the spreading of this virus. In this paper, research is made using the Industrial Internet of Things, deep learning, and a faster R-CNN algorithm to detect masked people and social distancing. The related existing research is discussed in this section.

In [9], an innovative intelligent technique based on a deep convolutional model is used to protect the people from COVID-19. The proposed system can detect automatically whether people are following the safety guidelines or not. In [8], a detailed comparison is done between the various deep learning approaches to monitor the disease from medical imaging. In another study [10], an IoT system based on temperature sensing, mask detection, and social distancing is suggested for the protection against COVID-19. Arduino Uno is used for the infrared sensing of temperature while Raspberry Pi is used for the mask detection and social distancing using computer vision techniques. In [11], an innovative mask identification technique is proposed by preprocessing of the image followed by face detection and image superresolution. The system is found to be very much accurate as far as identification of mask wearing is concerned. In [7], a model based on deep neural network is proposed for monitoring people and social distancing even in poor light conditions. The technique is found to be better than many other past techniques in terms of speed and accuracy. In [12], a computer vision based deep learning approach is used to examine the mask detection and social distancing automatically in real time. The model is developed on Raspberry Pi to monitor the different activities. It is found to save time and reduce the spread of COVID-19. In [13], an IoT-based deep learning foundation is provided for the detection of COVID-19. The provided model is used for the detection of a pandemic by applying this model on the X-ray of the chest. It is proved to be very accurate and hence quite helpful for the medical experts for the prompt detection of COVID-19. In [14], a technique based on open source computer vision is proposed to detect masked persons. The technique is found to be efficient especially in industrial applications.

In [15], the contribution of IoT and the concerned sensors for tracking and mitigating the virus is discussed. The study provides deep insight into e-health services based upon sensors for managing COVID-19 and discusses the subsequent IoT networks for the postpandemic era. In [16], a review of the technologies that may be used to detect COVID-19 is discussed. The review also discusses the future challenges in implementing these technologies. The technologies discussed are deep learning associated with X-ray, in vitro diagnostics (IVDs), and wearable sensors based on IoT for monitoring the COVID-19 patients. In [17], detection of the face wearing mask in the fine state has been done with context attention R-CNN technique with the help of special features for the purpose of region proposal and by dissociation of localization and classification fields. The context approach R-CNN has been found to be highly accurate. The authors in [18] have proposed a cellphone system-based detection system to extract four different kinds of features using the K-Nearest Neighbour algorithm. The system has been found to be excellent in terms of accuracy, precision, and recall. A system based on real time has been analyzed in [19] that uses the concept of generic detection. The accuracy of this system has been found to be high that may further be improved by including more parameters despite having a large computation time. Further in [20], a high-performance face mask detector based on deep learning has been proposed that is computationally less complex. The feature extraction has been improved by residual and regression modules.

Recently, many deep learning-based models have been developed for mask detection. The convolutional model is based on automatic recognition while the deep learning-based face mask detection technique is used for the high speed and accurate detection and feature extraction in a real-time environment even in bad light conditions. IoT-based models can be used for high accuracy detection while some systems based on superresolution image processing give extremely high accuracy. To overcome the gaps, the proposed system is based on IoT, deep learning, convolution, and automatic face recognition and computer vision to combine the advantages of all the techniques. Further, the proposed system uses the faster R-CNN technique to mitigate the effects of COVID-19.

## 3. The Proposed System Model

In this system, a model is suggested that uses the combination of OpenCV library with Raspberry Pi to build an Industrial Internet of Things (IIoT) application for mask detection and UAV application for social distance monitoring. The proposed system can identify or verify a person from a video frame. To see the masked face in a frame, first, we need to identify whether the facemask is present or not. If it is present, then it is marked as the region of interest (ROI) followed by its removal and processing for facial mask detection. The faster R-CNN algorithm for facemask detection works very well if the database contains clear images of persons. The employment of the OpenCV library tool proves to be very effective for mask detection and recognition.

### 3.1. Components of the Proposed Model

**Figure 1:**
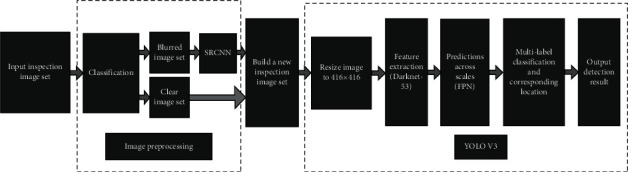
Constituents of the proposed model.

The various components of the proposed model are shown in Figure 1. A brief description of all the components is mentioned as follows.

#### 3.1.1. Image Classification

Currently, due to the worldwide epidemic, facial recognition has become more important in content-based video processing, such as pattern recognition, surveillance, computer vision, fraud detection, psychology, and neural networks. Facial recognition has attained a lot of consideration in research and the market [21, 22]. The facial detection technique is now used in the face-locking of cell phones, signing with face, etc. [23]. However, in some current situations, such as security circumstances and crowd flow assessment, the recognition of small and side faces is mandatory. So, the side faces and small faces pixels in the image are included. Currently, convolution neural networks (CNNs) [24, 25] have attained better outcomes in computer vision, such as recognition of an object and image labeling [26]. For this reason, the scientists also employ them to implement some algorithms in facial recognition [27], such as R-CNN, fast R-CNN, and faster R-CNN. It helps in achieving more accurate and quick results.

#### 3.1.2. Faster R-CNN Algorithm

This algorithm is composed of four main parts:

- Pretrained fully deep convolutional neural networks
- Region proposal network
- Region of interest (RoI) pooling and fully connected networks
- Bounding box regression and classification

In this research, firstly a deep convolutional neural network (CNN) is employed on a given image to produce feature maps that are given to the training classifier. The region proposal network uses two convolutional layers to identify the region. After that, ROI pooling is employed to collect and resize the feature maps in order to produce the new feature map. Finally, the classification and regression are applied to each region of the new feature map to predict the offset value for the bounding box. The object detection accuracy of R-CNN and fast R-CNN is very less than faster R-CNN. However, in the case of faster R-CNN, a separate network is employed to find the region of the proposal. As R-CNN is very slow and takes more computational time, therefore an advanced variant described as faster R-CNN [27] came into existence. To tackle the deviations of aspect ratio, faster R-CNN presents the concept of anchor boxes, which are employed to measure the ROIs. In terms of speed, faster R-CNN is 250 times faster than R-CNN [28].

#### 3.1.3. OpenCV

OpenCV is an open source library for machine learning. It was designed to give machine learning applications a common foundation and to increase the industrial usage of machine perception. Its library consists of over 2500 optimization algorithms which include classic and state-of-the-art machine learning and computer vision algorithms. Optimization algorithms [29–33] present in the libraries of OpenCV are used to identify faces, to perceive and categorize human motion in videos, to recognize objects, to track the camera movement, to retrieve 3D models, to make 3D point clouds using stereo cameras, to stitch images with one another to yield an image of high resolution, to find related images from an image dataset, to remove red eyes from images, and to review physical movement like eyes and hands. The vast range of applications of OpenCV includes stitching images of street view together, detecting face rapidly in Japan, detecting intrusions using video surveillance in Israel, creating robots that navigate and pick objects at Willow Garage, executing interactive art in countries like Spain and the United States of America, detecting drowning accidents in swimming pools across Europe, checking runways for debris in Turkey, and inspecting the products through labels in factories all around the globe. OpenCV supports many platforms like C++, MATLAB, and Java interface and all operating systems such as Linux, Windows, Mac OS, and Android [34].

The current research is targeted towards identifying the correct faces and hence providing the information to the user using OpenCV library.

#### 3.1.4. Calculation of Pixel Using Convolution Theorem

**Figure 2:**
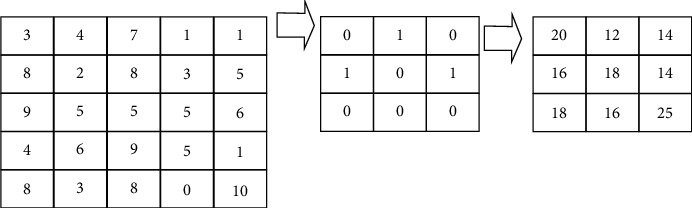
Convolution theorem.

The calculation of each pixel's intensity using convolution theorem is depicted in Figure 2. The mask above the arrow shows the filter used to calculate the convolution with the input image:

(a) In YOLO-v3, by using row and column matrix, output matrix can be obtained after applying the convolution as
  (1) $$\begin{array}{c} G\left(x,y\right)=\sum_{i}\sum_{j}h\left[j,k\right]\left[x+\left(-j\right),y+\left(-k\right)\right], \end{array}$$
where *h* denotes the input matrix as shown in Figure 2.
(b) Padding of images can be defined as
  (2) $$\begin{array}{c} p=\frac{\left(f-1\right)}{2}. \end{array}$$
Here, *f* is the filter dimension.
(c) Convolutional network can be defined as
  (3) $$\begin{array}{c} F\left(x\right)=\text{max}\left(0,x\right). \end{array}$$
(d) Final predicted classes can be obtained as
  (4) $$\begin{array}{c} C=\frac{e^{z_{i}}}{\sum_{j=1}^{K}e^{z_{i}}}. \end{array}$$
(e) Distance between feature can be computed as
  (5) $$\begin{array}{c} d=\sqrt{{\left(x_{i}-x_{j}\right)}^{2}+{\left(y_{i}-y_{j}\right)}^{2}}. \end{array}$$
Here, *i*, *j*=1,…, *N*, are directions.
(f) Pixel density ratio can be evaluated as
  (6) $$\begin{array}{c} \text{pdr}=\;\frac{R_{h}}{P_{h}}. \end{array}$$
Here, *R*_*h*_ shows the real height of the images in *mm*. In this paper, *R*_*h*_=1000. *P*_*h*_ represents the pixel height of the person in the frame in mm.
(g) Dimensions of the output matrix are given by
  (7) $$\begin{array}{c} N_{\text{out}}=\text{floor}\left(1+\frac{n+2p-f}{s}\;\right). \end{array}$$
Here, *n* is the image size. *f* is the filter size. *p* is padding and *s* is the stride, respectively.
(h) 3D matrix is given by
  (8) $$\begin{array}{c} \left[n,n,nc\right]*\left[f,f,nc\right]=\left[\text{floor}\left(1+\frac{n+2p-f}{s}\right),\text{floor}\left(1+\frac{n+2p-f}{s}\right),nf\right], \end{array}$$
where *nc* indicates the number of channels in the image and *nf* is the number of filters.
(i) Intermediate value is given by
  (9) $$\begin{array}{c} Z^{\left[1\right]}=W^{\left[1\right]}\cdot A^{\left[1-1\right]}+B^{\left[1\right]}A^{\left[1\right]}=G^{\left[1\right]}\left(Z^{\left[1\right]}\right). \end{array}$$
Here, *B* is biasing value. *A* is current single layer of 3D matrix image. *W* is the tensor filter and *G* is the intermediate value, respectively.
(j) Global pooling of the mean value is given by
  (10) $$\begin{array}{c} Q\left(i,j\right)=\frac{1}{d}\sum_{n=1}^{d}T_{n}\left(i,j\right). \end{array}$$
(k) Sigmoid function is given by
  (11) $$\begin{array}{c} \mathrm{SF}\left(x\right)=\left[\frac{1}{1+e^{-x}}\right]. \end{array}$$
(l) Pooling function is given by
  (12) $$\begin{array}{c} Q_{c}=\max\left(I_{c}\left(i,j\right)\right). \end{array}$$
(m) Average pooling function is given by

(13) $$\begin{array}{c} Q_{c}=\frac{1}{wh}\sum_{i=1}^{t}\sum_{j=1}^{w}I_{t}\left(i,j\right). \end{array}$$

#### 3.1.5. Industrial Internet of Things (IIoT)

Presently, IoT is a rapidly emerging technique in the industry context. It is the union of wireless networking, software processing, and computer networking. Internet of Things combines various physical things such as buildings, vehicles, and dissimilar devices embedded with intelligent sensors and allows these objects to exchange and collect data [4, 5, 35–39]. The aim of using IIoT is to share information and data all around the world. In addition, it provides automation by using various procedures to enhance our daily life [40]. IoT is an energetic worldwide wireless networking of daily objects connected to the Internet. IoT is the connection of virtual and physical things accessed via the Internet. A Wireless Sensor Network is used to connect sensors, in order to get data with a server or unique system to work. The aim of IoT is to connect the whole world through many intelligent places to automate, develop, and simplify human life [41]. Currently, UAVs are thought to be excellent remote sensing technologies for collecting information over larger surfaces. UAV tools as sensing tools are now being used in the industry sectors for solving and avoiding various problems. UAVs are extremely reliable high-tech platforms for effective and economical information gathering and event monitoring. IIoT transmits the information from systems that examine and command the physical world to data managing systems that intelligent cloud computing has presented to be an essential platform for meeting the data handling constraints.

Our research introduces an intelligent IIoT remote sensing of face detection and control system based on UAV. The IOT gateway provides automatic integration of UAV into an industrial system, while UAV photographs are scientifically and instantaneously calculated and examined in the cloud [42].

#### 3.1.6. Unmanned Aerial Vehicle

An unmanned aerial vehicle (UAV) or drone is one of the unmanned vehicles that carry a payload from one place to another place. Several types of aerial vehicles have been designed and developed to keep up with the times while integrating all the innovative technologies like self-balancing, autoflight/pilot mode, obstacle detection, and long-range and long-time battery backup [43]. This is a great attempt to implement an intelligent modular unmanned aerial vehicle that can be convenient in different environments where manpower is not reachable or challenging due to some physical barriers. Besides, it also tries to reduce cost and complexity. Surveys in the area of unmanned aerial vehicles have been significantly conducted but modular functionality in UAVs is yet to be developed. Modularity can create multipurpose usage capabilities and can execute unique tasks with different and standalone swappable units. There are many illustrations in day-to-day human life where the modular technique of UAV can be handy. Drones are used as telemedicine in telecommunication technology [44]. Drones are used to communicate with patients and gather their information and assist telemonitoring in remote areas [45].

In this research paper, a UAV primarily used for aerial photography can swap modules to have an amphibious landing and Raspberry Pi kit for real-time image recognition. It can be implemented in real life for data collection research, aerial survey, security provision, and social distance monitoring in crowded places [46–48]. The drone can easily detect the distance between persons and objects and also chairs, birds, dogs, trees, grass, buildings, etc. It reports as a warning to the administration and higher priority people.

#### 3.1.7. Raspberry Pi

It is an economical device just like a computer through which programming is implemented using Python. Raspberry Pi 4 is used in the current research work as it offers more memory capacity and a better interface. The current model is the fastest and most powerful model of Raspberry Pi. Python is used for the programming of Raspberry Pi 4 in the proposed work. Raspberry Pi 3/Raspberry Pi 4 is one of the IIoT boards for the detection of objects in real time. This is an IoT motherboard with Wi-Fi.

### 3.2. The Proposed Methodology

**Figure 3:**
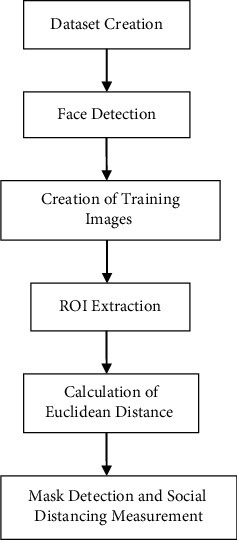
Flow process of the proposed methodology.

The algorithm used in the script is faster R-CNN. The flow process of the proposed methodology is shown in Figure 3. The steps are mentioned as follows:

1. Dataset of images is taken for mask detection after running database_create.py code.
2. The faces in image/video stream are detected.
3. The training images are automatically created in database folder (with YOLOv3) that contains the images of the masked face to be recognized. While creating the database, image loss is also considered.
4. The region of interest (ROI) is extracted for each face with two vectors (i.e., automated test maintenance time and automated test analysis time) per ROI with regression effects. ROI is pipelined and pooled with feature mapping. In ROI pooling, ROI is taken from the input and a portion of the input feature map for that particular ROI is taken and that feature-map portion is transformed into a fixed dimension map.
5. The Euclidean distance [49] is calculated between 2 ROI points and hence the centre coordinates are transformed into rectangular coordinates to make the bounding box.
6. The bounding box is used to detect the number of people with or without mask and measure the social distancing. For this, face_det.py script is used. The YOLO detector in association with darknet [50] is used here for social distancing and object recognition.

**Figure 4:**
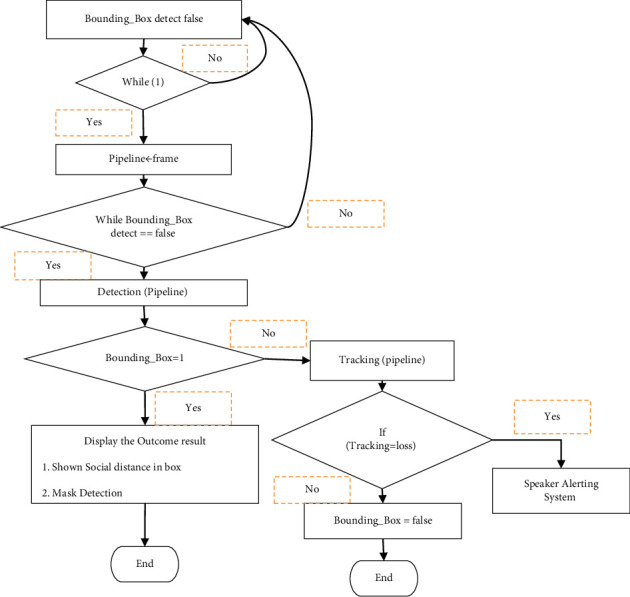
Pseudocode for detection of bounding box using OpenCV camera.

The pseudocode for detection of bounding box using OpenCV camera is shown in Figure 4.

## 4. Results

The simulation is performed for 18000 training images collected in the various situations. The validation of the trained model is done against the testing set of 8811 images obtained after simulation on PyTorch. The various parameters such as validation loss, validation accuracy, precision, recall, and F1-score are also calculated in this section. The comparison of the proposed technique for the various situations is also done with other existing state-of-the-art techniques applied on the same image set. Finally, the results for unmask/mask faces, social distancing, and count of persons with real-time video streaming are also presented.

**Figure 5:**
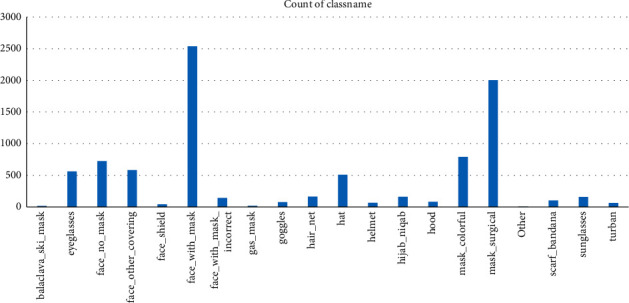
Count of the testing dataset in various classnames.

The various classnames in the dataset with the count of training dataset and testing dataset are shown in Table 1. The count of the various categories in testing dataset is also shown in Figure 5.

### 4.1. Validation Loss

The training loss is the error for the training data set (i.e., it is measured during each epoch). On the other hand, the validation loss is the error after passing the validation data through the network already trained (i.e., it is measured after each epoch). An epoch refers to the number of passes of the complete training dataset. The validation loss may be calculated from the training as

(14) $$\begin{array}{c} \text{validation Loss}=\frac{\text{training loss}}{\text{training images}}\times 1000. \end{array}$$

Training loss and validation loss are never measured in percentage. Rather, they are measured in terms of the mean square value of error.

**Figure 6:**
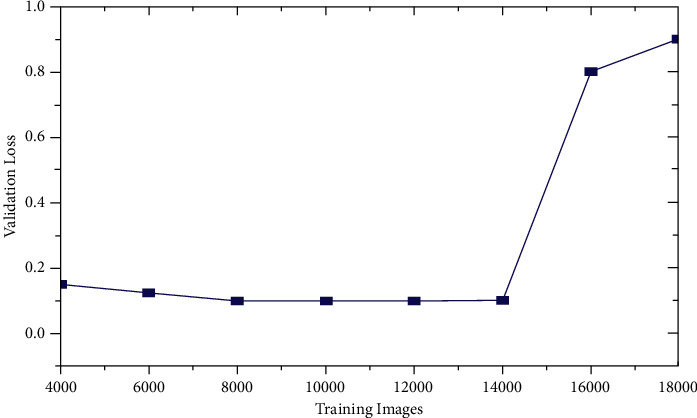
Variation of validation loss with training models.

The training loss and the validation loss for the various training images are shown in Table 2. It may be observed that the validation loss remains constant or increases as the training loss improves from 14000 images set to 4000 images set. This condition is referred to as overfitting. Beyond 14000 training images, everything is fine as both are progressing in the same direction. Further, Figure 6 shows the variation of the validation loss with respect to the number of training images. As the number of training images is increased, the validation loss first decreases at a small rate but after reaching 14000 training images, the validation loss starts increasing at a much steeper slope.

### 4.2. Validation Accuracy

**Figure 7:**
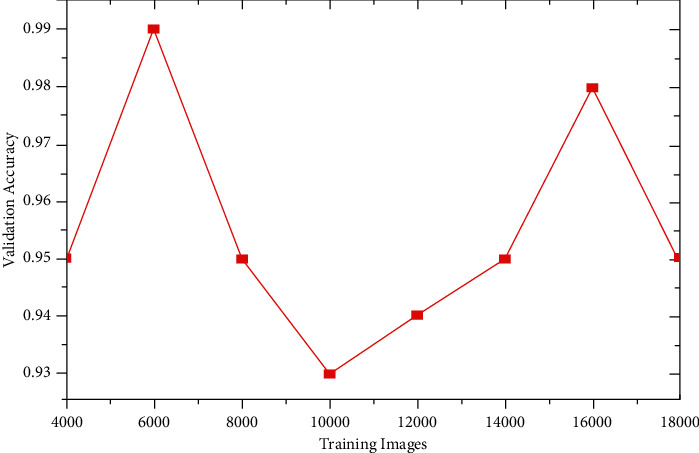
Variation of validation accuracy with training models.

The validation accuracy is an important metric used to predict the performance of an algorithm. The model is validated by calculating both metrics–loss and accuracy. The validation accuracy against each value of validation loss for the different training images is shown in Table 3. The last column shows that the validation accuracy is always less than 1 and it is expressed as a percentage after multiplying that value by 100. It may be observed that the validation accuracy fluctuates as the validation loss is decreased by increasing the number of training images from 4000 to 14000. So, this portion is referred to as overfitting (as shown earlier). Beyond that, if we increase the number of training images from 16000 to 18000, the accuracy decreases with the increase in loss. It means that the model is learning correctly.  Figure 7 shows the variation of the validation accuracy with the increase in the number of training images.

### 4.3. Precision, Recall, and F1-Score

The precision and recall values are calculated with the help of True Positives (TP), False Positives (FP), True Negatives (TN), and False Negatives (FN) [51]. Precision indicates the ratio of positive observations predicted correctly to the total number of positive observations predicted, while recall refers to the ratio of positive observations predicted correctly to the total number of observations.

(15) $$\begin{array}{c} \text{Precision}=\frac{\text{TP}}{\text{TP}+\text{FP}},\\ \text{recall}=\frac{\text{TP}}{\text{TP}+\text{FN}}. \end{array}$$

F1-score is the harmonic mean of the recall and precision.

Table 4 shows the precision, recall, and F1-score for the testing dataset where dataset “with mask” corresponds to the datasets corresponding to the sr. nos. 1, 6, 15, and 16 of Table 1 and “without mask” corresponds to all the remaining datasets.

Table 4 shows that the precision, recall, and F1-score are excellent for both types of datasets.

### 4.4. Comparison with Existing Techniques

The proposed technique, i.e., faster R-CNN, is compared with the other techniques applied on the same dataset. The comparison is shown in terms of speed (frames/sec) in Table 5. Category 1, Category 2, Category 3, and Category 4 refer to the college campus, traffic area, street, and shopping mall. The proposed technique faster R-CNN is found to be better than the other techniques GravNet approach and DGCNN approach [52].

### 4.5. Visual Analysis

Most of the AI and deep learning programming is inbuilt in a drone for the higher speed of operation and higher accuracy. Modern drones capture aerial photography with real-time constraints. Real-time computation time is very less while using drones. OpenCV, PyTorch, tensor flow, and Keras packages are required for solving and recorrecting video stream stability problems and helping in object detection problems.

**Figure 8:**
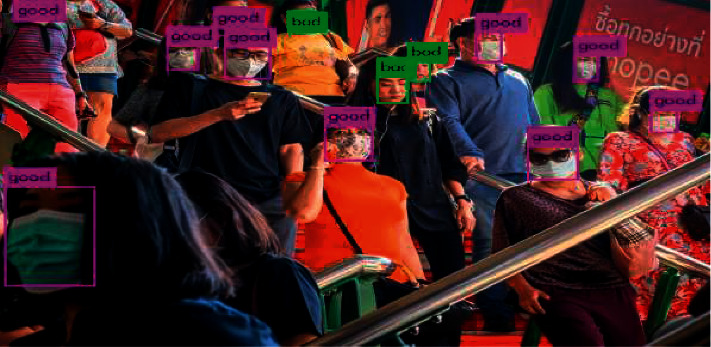
Mask count (pink colored) and unmask count (green colored).

**Figure 9:**
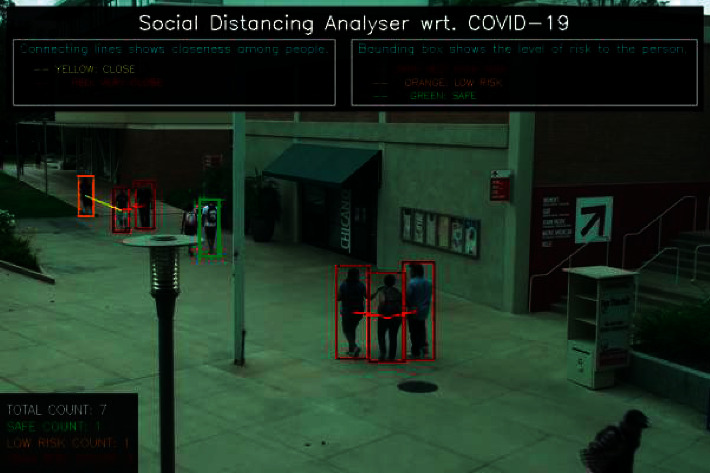
Social distance maintenance and green/orange/red object detection.

In Figure 8, mask count and unmask count are shown using the benchmark mask dataset [53]. The dataset is trained in the first stage, the image is detected using the faster R-CNN algorithm in the second stage, and video objects are detected with the counting of persons in the third stage. The social distancing majorly calculates the objects of video streaming per pixel. A surveillance system is supported and automated to monitor the social distance count using OpenCV and faster R-CNN algorithms in drone surveys when wireless protocols are used. The system detects a number of persons visiting an area who are at higher risk.  Figure 9 shows the social distance detection and the experiment performed on the benchmark birds-eye video dataset [54]. The green, orange, and red objects indicate the objects having safe distancing, marginal distancing, and unsafe distancing, respectively.

**Figure 10:**
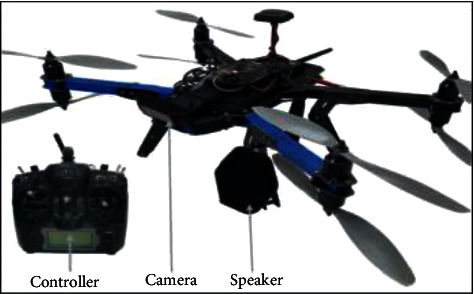
Overview of the drone monitoring system components.

Figure 10 shows the overview of the drone system. The speaker alerting system is programmed with a condition referred to as “when the distances between the objects are less than 1 meter” in the IoT controller. After satisfying the condition, the recorded voice sounds are activated to be disposed of the crowd. This system is used only to alert the people to maintain social distancing and inform them to wear masks. The outcome result of this method is based on the recorded voice output and monitoring output through IoT clouding.

The drone mechanism includes an IoT controller and a high-fidelity camera. Automatically, the speaker announces whenever the condition is solved.

## 5. Conclusion

The drones can be used to detect a group of people who are unmasked and do not maintain social distance. In this paper, a drone was used for detecting a number of objects using a faster R-CNN algorithm and YOLOv3. Initially, faster R-CNN was used for mask detection. Raspberry Pi 4 interfaced OpenCV camera in a real test case was implemented to capture the images. The built face classification was stored on the cloud; thus the built drone always remains connected with the cloud server using the Internet. YOLOv3 model was used for measuring the social distance. Faster R-CNN and YOLOv3 were utilized since they can easily calculate the captured images with better performance in a few milliseconds. Extensive results were drawn for social distancing, unmask/mask faces, and count of persons with real-time video streaming. PyCharm software was utilized using Python for reducing the cost of the system. Extensive comparative analyses revealed that the proposed model outperforms the competitive models in terms of various performance metrics.

In this paper, no novel deep learning model was proposed. Therefore, in near future, we will design novel deep learning models to achieve better results. Also, the proposed model can be extended for indoor application areas too.

## Figures and Tables

**Table 1:** Classnames in the dataset with images divided into training and testing dataset.

S. no.	Classname	Count of training classname (training dataset)	Count of testing classname (testing dataset)
1	balaclava_ski_mask	31	15
2	Eyeglasses	1150	563
3	face_no_mask	1481	724
4	face_other_covering	1192	583
5	face_shield	88	43
6	face_with_mask	5183	2539
7	face_with_mask_incorrect	291	142
8	gas_mask	37	18
9	Goggles	153	75
10	hair_net	334	163
11	Hat	1038	508
12	Helmet	137	67
13	Hijab_niqab	331	162
14	Hood	165	81
15	mask_colorful	1618	792
16	mask_surgical	4093	2004
17	Others	12	6
18	Scarf_bandana	210	103
19	Sunglasses	325	159
20	Turban	131	64
**Total**		**18000**	**8811**

**Table 2:** Training loss and validation loss for the training images.

S. no.	Training images	Training loss	Validation loss
1	4000	0.6	0.15
2	6000	0.75	0.125
3	8000	0.8	0.1
4	10000	1	0.1
5	12000	1.2	0.1
6	14000	1.4	0.1
7	16000	12.8	0.8
8	18000	16.2	0.9

**Table 3:** Validation accuracy for the training images.

S. no.	Training images	Validation loss	Validation accuracy
1	4000	0.15	0.95
2	6000	0.125	0.99
3	8000	0.1	0.95
4	10000	0.1	0.93
5	12000	0.1	0.94
6	14000	0.1	0.95
7	16000	0.8	0.98
8	18000	0.9	0.95

**Table 4:** Precision, recall, and F1-score for testing dataset.

Dataset	Precision	Recall	F1-score
With mask	0.99	0.86	0.92
Without mask	0.88	0.99	0.93

**Table 5:** Comparison (in terms of speed in fps) of faster R-CNN with other techniques.

Technique	Category 1	Category 2	Category 3	Category 4
Faster R-CNN	40.9	50.5	11.2	25.5
GravNet	61.9	56.1	14.4	28.4
DGCNN	89.8	90.2	47.9	62.4

## Data Availability

The data used to support the findings of this study are available from the corresponding author upon request.
